# Communication about weight‐related issues with adult patients with obesity in general practice: A scoping review

**DOI:** 10.1002/osp4.669

**Published:** 2023-04-10

**Authors:** Cecilie Sonne Lindberg, Annelli Sandbaek, Sissel Due Jensen, Jens Meldgaard Bruun, Pernille Andreassen

**Affiliations:** ^1^ Steno Diabetes Center Aarhus Aarhus University Hospital Aarhus Denmark; ^2^ Research Unit for General Practice Aarhus Denmark; ^3^ Danish National Center for Obesity Aarhus Denmark; ^4^ Department of Public Health University of Aarhus Aarhus Denmark; ^5^ Department of Clinical Medicine University of Aarhus Aarhus Denmark

**Keywords:** communication, obesity, primary care, scoping review

## Abstract

**Background:**

Primary care providers see patients with obesity in general practice every day but may be challenged regarding communication about obesity. The research question of this study is: how do general practitioners and general practice staff and adult patients with obesity communicate about weight‐related issues?

**Methods:**

A scoping review approach was used, searching PubMed, Scopus and CINAHL for peer‐reviewed studies – of both quantitative and/or qualitative study designs, and published between 2001 and 2021.

**Results:**

Twenty articles were included. The weight‐related issues discussed were by far physical issues, and only one study mentioned psychosocial issues. Most of the included studies contained information on who initiates the communication, how the weight‐related issues are addressed and handled, and also obstacles and challenges in relation to the communication. The studies lacked information of when the weight‐related issues are addressed and differences in views and experiences when discussing weight‐related issues in general practice.

**Conclusion:**

Studies with the main focus communication about obesity and overall health in general practice are needed. Findings also indicate, that non‐stigmatizing communication tools and guidelines are needed on this area to promote these types of conservations.

## 1 INTRODUCTION

### 1.1 Rationale

The number of people living with obesity continues to increase worldwide, affecting world health in a negative manner.^1^, ^2^, ^3^, ^4^ In most Western countries, a well‐established primary care system has been developed^5^ and many adults with obesity visit primary care providers, such as general practitioners and general practice staff, every day, dealing either directly or indirectly with weight‐related issues, making these consultations an obvious possibility for addressing the topic. This could for instance be a patient with obesity and a musculoskeletal complaint, type II diabetes, cardiovascular disease, cancer, depression or eating disorder.

Patient‐provider communication has shown to play an important role in the doctor‐patient meeting and shared decision making^6^ has shown to have the potential to promote an appropriate conversation about weight between patients and health care providers.^7^ It is well known that health professionals struggle to communicate with patients about the topic of overweight and obesity, for example, by not effectively communicating the diagnosis of obesity,^8^, ^9^ and much of the literature focuses on weight stigma as a huge problem in clinical settings.^10^, ^11^ But how do general practitioners and general practice staff and adult patients with obesity communicate about weight‐related issues—and which types of weight‐related issues are addressed? No review has sought to gather and explore studies to make clarification of the existing literature on this subject and also identify knowledge gaps in the research on this topic which underpins the need for this review. Several reviews have focused on the management of obesity in general practice, but none focusing on communication about weight‐related issues.^9^, ^12^, ^13^, ^14^, ^15^, ^16^, ^17^, ^18^, ^19^, ^20^ Because of the diversity and complex nature of the topic of communication, and because of the limited knowledge on the topic, a scoping review approach was found most suitable.^21^

### 1.2 Objectives

The main review question of this scoping review is: *What is known about communication about weight‐related issues in general practice with adult patients with obesity?* Also, the review seeks to examine the different types of weight‐related issues that are addressed in the included studies. The review explores when the weight‐related issues are being addressed, by whom and how in the relevant studies. Furthermore, the way in which weight‐related issues are handled, differences in experiences, and obstacles and challenges when communicating obesity are examined. The abovementioned will lead to the determination of knowledge gaps for future studies.

## 2 METHODS

The five stages of a scoping review were followed.^22^, ^23^ Also, this scoping review was based on the Joanna Briggs Institute Manuel for scoping reviews^21^ and the Preferred Reporting Items for Systematic reviews and Meta‐Analysis extensions for Scoping Reviews.^24^

### 2.1 Protocol and registration

To ensure transparency and reduce the risk of duplication, an a priori protocol was developed with information of the objectives, inclusion criteria and methods. The protocol was uploaded immutable and time‐stamped on Open Science Framework Registries on 3 December 2021 with the online link: https://osf.io/kv42z/?view_only=a2abd427531d407fb575129f7071c732.

### 2.2 Eligibility criteria

The making of the eligibility criteria took its starting point in the “PCC” mnemonic consisting of the Population, Concept, and Context.^21^ In this review, the population is adult people living with obesity, the concept is communication about weight‐related issues, and the context is general practice. An overview of the definitions and eligibility criteria of this scoping review is presented in Table 1.

**TABLE 1:** Overview of definitions and eligibility criteria of this scoping review.

PCC	Theme	Definition in this scoping review	Inclusion criteria	Exclusion criteria
Population	Adults	≥18 years	Adults people (≥18) years, both sexes.	Only concerning children in general (age in years <18)
	Obesity	Use of World Health Organization definition of obesity for adults^1^ ^,^ ^a^	People with BMI ≥30	Only concerning people with BMI <30^c^
		Overweight: BMI^b^ ≥ 25		
		Obesity: BMI ≥ 30		
Concept	Communication	Verbal communication means both oral and written communication^25^	Dealing with verbal communication.	Not dealing with communication as a topic or only focusing on nonverbal communication.
	Weight‐related issues	Weight‐related issues in the review refers to both physical and psychosocial issues.^26^, ^27^, ^28^	Dealing with weight‐related issues as topic.^c^	Not dealing with weight‐related issues as a topic.
Context	General practice/general practitioner/general practice staff	The terms used for general practice, general practitioners and general practice staff shows great diversity and overlap internationally.^5^, ^29^	In general practice from all over the world. General practitioners and all general practice staff.	Not in general practice.
		In Table 2 of this study total transparency is used concerning which terms of general practitioners and general practice staff are extracted from the studies.		
Not relevant	Study design	Not relevant	Published peer‐reviewed quantitative, qualitative and mixed methods study designs, original research articles only.	Case reports, reviews, “gray literature,” for example, conference abstracts, dissertations, and unpublished studies.
	Publication period	Not relevant	≥2001	<2000
	Language	Not relevant	Written in English	Written in another language than English

^a^ The WHO definition of BMI is used for people 20 years and older. To be pragmatic, this review defines adults as 18 years or older.

^b^ BMI, Body Mass Index.

^c^ In the a priori protocol, it is mentioned that people with BMI <30 are excluded from the review. When conducting this review, it was clear that patients with overweight often could not be clearly separated from the group of patients with obesity, and therefore this scoping review also included studies dealing with both patients with overweight and obesity.

### 2.3 Information sources and search

To ensure access to a diversity of studies with both quantitative and/or qualitative study designs, the online databases PubMed, Scopus, and CINAHL were searched. All final searches were conducted on Monday 6 December 2021. The before mentioned online available review protocol shows an example of a complete search on PubMed. For each of the included studies for data extraction, the reference lists were screened for additional studies for inclusion.

The search strategy for all three databases was developed in cooperation with a health science librarian, and the final search strategy was examined by another health science librarian.

### 2.4 Selection of sources of evidence

All records were uploaded to EndNote and afterward Covidence where the duplicate removal tools were used by both programs. The screening was done by the first and senior author using Covidence and performed independently by two reviewers with different professions. Titles and abstracts were independently read and assessed in the first round of exclusion, based on the predefined inclusion criteria pre‐specified in the review protocol. The remaining studies which seemed to meet or maybe meet the predefined criteria were read in full‐text in the second round of exclusion. Reasons for exclusion of full‐text papers not meeting the inclusion criteria were recorded for use in the flowchart. After the second round of exclusion, reference list and studies found when making the initial searches were looked through, and no additional studies were found relevant by agreement of the two authors doing the screening.

### 2.5 Data charting process and data items

Data extraction was done by the first author using the data extraction tool developed by the authors made by modifying the standardized tool made by Joanna Briggs Institute (available in the review protocol^21^). The data charting included citation details, origin/country of origin, aim(s), study design, participants' details, methodology and main findings related to the scoping review questions(s). Weight‐related issues were only extracted from the studies if they were mentioned in relation to communication of weight‐related issues and not if they were only mentioned in for example, a demographic table describing the population. The data charting also consisted of a qualitative descriptive content analysis approach to extract the data about the communication.

### 2.6 Critical appraisal of individual sources of evidence

The validated tool for assessment of study quality, the Quality Assessment Tool for Studies with Diverse Designs (QATSDD),^30^ was used for the quality assessment because of the suitable fitting for both quantitative, qualitative and mixed‐method studies. The QATSDD uses a 16 item scoring system with a Likert scale (0–3 points) for each item.

### 2.7 Synthesis of results

The data were analyzed and presented as described in the protocol. Also, the descriptive and numerical analysis and summary table with information on the extent and nature of the included studies was developed, and in relation to this the weight‐related issues were further categorized into greater categories, making it more accessible. A table with a presentation of the quality assessment score was developed.

## 3 RESULTS

### 3.1 Existing studies on the topic

**FIGURE 1:**
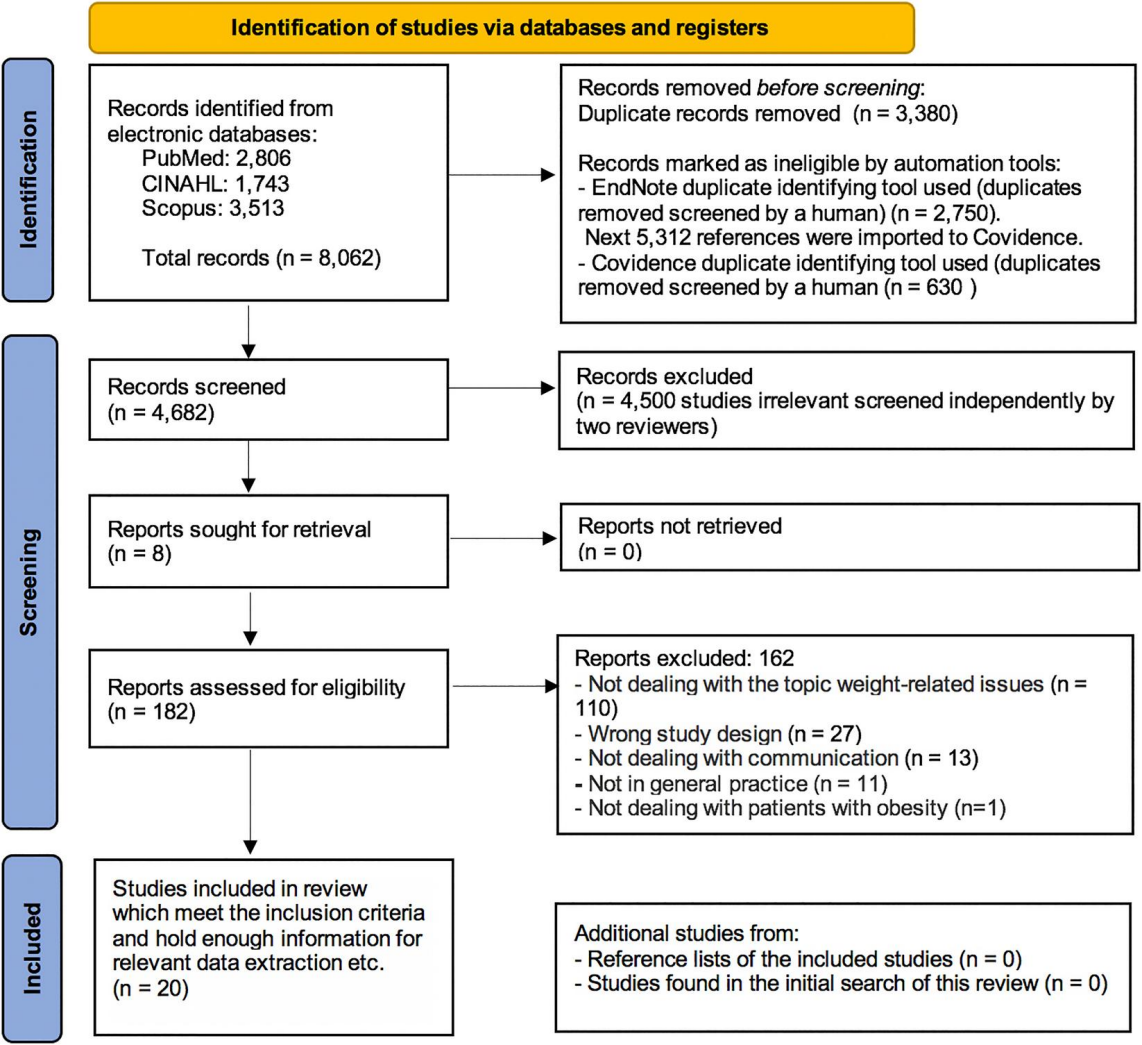
Flow diagram. All screening and inclusion of studies presented in the Preferred Reporting Items for Systematic Reviews and Meta‐analyses (PRISMA) flow diagram,^31^ which is automatically made by Covidence, was downloaded and hereafter adjusted manually to fit the approach of this scoping review.

A total of 8062 records from PubMed, CINAHL and Scopus were identified, and after using different methods for duplicate removal, 4682 records were screened on title/abstract level by the two reviewers. 182 articles were screened for eligibility by the same two reviewers by reading full text. This resulted in 20 articles qualifying for data extraction etc (Figure 1). Tables 2 and 3 respectively give an overview of the study characteristics and descriptive numerical analyses and summary of the 20 included studies. The majority of the studies were published after 2014 (*n* = 13, 65%). As regarding study design, 12 studies were qualitative (60%), 5 mixed‐method (25%) and 3 quantitative (15%). Most of the studies were conducted in USA (*n* = 10, 50%) and secondly in United Kingdom (*n* = 3, 15%). All of the included studies dealt with general practitioners (*n* = 20, 100%). Only a very small proportion of the included studies focused on a specific patient subgroup (*n* = 3, 15%). The majority of the studies dealt with both overweight and obesity (*n* = 14, 70%), and the rest of the studies solely dealt with obesity (*n* = 6, 30%). Because of the great amount and diversity of weight‐related issues, the authors have categorized these items in major categories in Table 3, which reveals that the most frequent categories mentioned in the studies were “impact on general health” (*n* = 18, 90%), “diabetes and prediabetes” (*n* = 15, 75%), “hypertension and impact on blood pressure” (*n* = 14, 70%), “hyperlipidemia and affected blood lipids” (*n* = 8, 40%) and musculoskeletal problems (*n* = 8, 40%). Only one study held information of psychosocial (i.e., depression) weight‐related issues (*n* = 1, 5%). The study characteristics regarding the subdivisions of communication of this review will be described in details in Section 3.3.

**TABLE 2:** Overview of the study characteristics of the included studies.

Citation details	Study aim (s)	Country	Study design	Participants details	Methodology	Main findings
Reference number ^32^: Title: Obesity in general practice: a Focus group study on patient experiences Author(s): Kirsti Malterud and Kjersti Ulriksen Date of publication: Dec 2010 Journal, volume, issue, pages: Scand J Prim health care Dec 2010; 28(4):205–10	Exploring patients with obesity's experiences with GPs management of their weight issues.	Norway	Qualitative	Sample size: 13 Age: 30–55 years Sex: 8 female and 5 male Geographical location: Norway GPs or/and GP staff subgroup(s): General practitioners (GPs) Patients with obesity subgroup(s): NA Patients in the study only with obesity or both overweight and obesity included: Only patients with obesity Types of weight‐related issues: Diabetes, blood pressure, back problems, knee problem, urinary problems, obesity side effects, additional weight‐related diseases	Focus‐group interviews	The care of obesity can be improved if GPs introduce the topic of obesity to patients in a sensitive way. The topic of obesity should be introduced by maintaining medical attention to problems, adequate follow‐up, knowledge about service resources and prevention of blaming.
Reference number ^33^: Title: Do primary care physicians use the 5 As in counseling obese patients? A qualitative study Author(s): Jean‐Jasmin Mi‐Li Lee, Yew Cheong Tung, Kit Ping Tai, Samantha Alexis Tay, Shu Juan Cheng, Seng Bin Ang and Ngiap Chuan Tan Date of publication: 12 January 2017 Journal, volume, issue, pages: Proceedings of Singapore healthcare 2017; 26(3):144–149	To explore primary care physicians' modalities in obesity consultation and correspond to the 5 As communication tool.	Singapore	Qualitative	Sample size: 50 Age: 26–61 years Sex: 21 female and 29 male Geographical location: Singapore GPs or/and GP staff subgroup(s): Primary care physicians (PCPs)—Private general practitioner (N 30) or Polyclinic doctor (Dr) (N 20) Patients with obesity subgroup(s): NA Patients in the study only with obesity or both overweight and obesity included: Both patients with overweight and obesity Types of weight‐related issues: Diabetes, hypertension, hyperlipidemia, obstructive sleep apnea, impaired glucose tolerance	Focus group discussions and in depth interviews	The obesity consultation methods of primary care physicians' varied depending on context and setting. Regarding the 5 As, “Ask,” “assess” and “Advise” were more often used than “Agree” and “Arrange.”
Reference number ^34^: Title: A qualitative inquiry about weight counseling practices in community health centers Author(s): Gillian L. Schauer, Rebecca C. Woodruff, James Hotz, Michelle C. Kegler Date of publication: 12 June 2014 Journal, volume, issue, pages: Patient education and counseling, 2014, Vol. 97 (1), p. 82–87	To explore how clinicians approach weight counseling in adults.	USA	Qualitative	Sample size: 30 Age: 18–65 Sex: 14 male and 16 female Geographical location: Georgia, USA GPs or/and GP staff subgroup(s): Primary care physicians and physician assistants and nurse practitioners Patients with obesity subgroup(s): NAPatients in the study only with obesity or both overweight and obesity included: Both patients with overweight and obesity Types of weight‐related issues: Weight‐related chronic or comorbid conditions, obesity related issues, diabetic, diabetes, blood pressure out of control, high blood pressure, depression, benefit to their health, blood sugar is up, heart attack, stroke, kidney disease	Semi‐structured interviews	A variety of the approaches were used to address weight and many of these are not evidence‐based. A linkage with weight loss resources in the health care system or community are not widely reported.
Reference number ^35^: Title: Weight communication: How do health professionals communicate about weight with their patients in primary care settings? Author(s): Stephanie Aboueid, Rukhsana Ahmed, Monika Jasinska, Catherine Pouliot, Billie Jane Hermosura, Ivy Bourgeault & Isabelle Giroux Date of publication: 14 December 2020 Journal, volume, issue, pages: Health communication, 2020‐12‐14, p. 1–7	To investigate how health professionals (HPs) communicate with patients about weight.	Canada	Qualitative	Sample size: 33 Age: NA Sex: 29 female and 4 male Geographical location: Canada GPs or/and GP staff subgroup(s): 7 family physicians and 13 nurse practitioners and 13 dietitians Patients with obesity subgroup(s): NA Patients in the study only with obesity or both overweight and obesity included: Both patients with overweight and obesity Types of weight‐related issues: Out of range blood markers, pain, type 2 diabetes, side effects on overall health, diabetes, high blood pressure, obesity‐related chronic diseases, hypertension, high cholesterol	Semi‐structured interviews	Communication about weight can be a sensitive area and many different approaches are used depending on patients and HP factors. Future guidelines could possibly benefit by shifting to communication about modifiable risk factors rather than weight.
Reference number ^36^: Title: Speaking of weight: How patients and primary care clinicians initiate weight loss counseling Author(s): John G. Scott, Deborah Cohen, Barbara DiCicco‐Bloom, A. John Orzano, Patrice Gregory, Susan A. Flocke, Lisa Maxwell, and Benjamin Crabtree Date of publication: 27 February 2004 Journal, volume, issue, pages: Preventive medicine 2004, Vol. 38 (6), p. 819–827	To characterize how talking about weight is constructed and aiming for identifying strategies that might increase the frequency of consultations about weight loss in primary care.	USA	Mixed method	Sample size: 327 adults (age >20) and 49 children (age ≥2 and ≤20) 49 Age: NA Sex: 204 female and 172 male Geographical location: Nebraska, USA GPs or/and GP staff subgroup(s): Primary care physicians Patients with obesity subgroup(s): NA Patients in the study only with obesity or both overweight and obesity included: Both patients with overweight and obesity Types of weight‐related issues: High blood pressure, high cholesterol, out of control diabetes, back pain, heartburn, and leg swelling, chronic conditions, weight‐related issues, elevated blood pressure, hypertension	Descriptive field notes	The strategies that increase the possibility of patients identifying weight as a problem, or that provide clinicians with a way to ‘‘medicalize’’ the patient's obesity, are likely to increase the frequency of weight loss counseling in primary care visits.
Reference number ^37^: Title: Current attitudes and practices of obesity counseling by health care providers Author(s): Christine Petrin, Scott Kahan, Monique Turner, Christine Gallagher, William H. Dietz Date of publication: 25 August 2016 Journal, volume, issue, pages: Obes Res Clin Pract. May–Jun 2017; 11 (3): 352–359	To explore the attitudes and practices of health care professionals (HCPs) regarding obesity counseling.	USA	Quantitative	Sample size: 1501 Age: 46,5 (mean) Sex: male 62% and female 38% Geographical location: United States GPs or/and GP staff subgroup(s): Primary care physicians (PCPs) (internists and family physicians) and nurse practitioners (NPs) Patients with obesity subgroup(s): NA Patients in the study only with obesity or both overweight and obesity included: Both patients with overweight and obesity Types of weight‐related issues: Obesity‐related diseases, obesity‐related risk factors, risk of heart disease, increased blood pressure, complications of diabetes, abnormal lipids, poor glycemic control	Web‐based survey	The research gives insight in the current attitudes and practices of a range of HCPs. There are both areas of agreement and consistency across specialties. There are instances where certain providers might be counseling more effectively than others. The need for better education and role modeling is a main conclusion—particularly in the area of appropriate language.
Reference number ^38^: Title: Enabling tomorrow's doctors to address obesity in a GP consultation: An action research project Author(s): Kathleen E. Leedham‐Green, Rebecca Poundband Ann Wylie Date of publication: 14 July 2016 Journal, volume, issue, pages: Educ Prim care. 2016 Nov; 27(6):455–461	To give insight in how senior medical students consult with patients with obesity in general practice, show the range of learning needs, and the impact of various educational strategies that aim to bring their practice closer to current evidence‐based guidelines.	United Kingdom	Qualitative	Sample size: 305 Age: NA Sex: NA Geographical location: London, United Kingdom GPs or/and GP staff subgroup(s): General practitioners (GPs) and senior medical students in general practice Patients with obesity subgroup(s): NA Patients in the study only with obesity or both overweight and obesity included: Only patients with obesity Types of weight‐related issues: Hypertension, diabetes, risk factors of obesity	Action research	A systematic identification and addressing of learning needs, including barriers and enablers to best practice on how senior medical students and their GP tutors can acquire the role of legitimacy and role of competency required for effective practice was conducted in the study.
Reference number ^39^: Title: Overcoming obesity: A mixed methods study of the impact of primary care physician counseling on low‐Income African American women who Successfully Lost weight Author(s): Elaine Seaton Banerjee, MPH, Sharon J. Herring, Katelyn E. Hurley, Katherine Puskarz, Kyle Yebernetsky, and Marianna LaNoue Date of publication: 16 March 2017 Journal, volume, issue, pages: Am J health promot. 2018 Feb; 32(2):374–380	To have insight in the interactions between low‐income, African American women who successfully lost weight and their primary care physicians (PCPs).	USA	Mixed method	Sample size: 71 Age: 43 (control) and 44,9 (case) both mean Sex: Women Geographical location: Philadelphia, USA GPs or/and GP staff subgroup(s): Primary care physician Patients with obesity subgroup(s): Low‐Income African American women who Succesfully lost weight Patients in the study only with obesity or both overweight and obesity included: Both patients with overweight and obesity Types of weight‐related issues: Diabetes, impact of weight in health problems, problem of obesity in relation to other health problems, blood pressure out of control	Surveys and interviews	PCP counseling may be an important factor in promoting weight loss in low‐income, African American women. Patients may benefit from their PCPs drawing connections between obesity and weight‐related medical conditions and enhancing intrinsic motivation for weight loss.
Reference number ^40^: Title: Primary care providers' communication with patients during weight counseling: a Focus group study Author(s): Kimberly A. Gudzune, Jeanne M. Clark, Lawrence J. Appel, and Wendy L. Bennett Date of publication: 21 July 2012 Journal, volume, issue, pages: Patient Educ Couns. 2012 Oct; 89(1):152–7	Use focus groups and qualitative methods to explore primary care providers (PCPs)’ usual practices as part of weight counseling to identify how PCPs communicate with their patients about weight loss.	USA	Qualitative	Sample size: 26 Age: mean 46, Sex: 15 female Geographical location: Maryland, USA GPs or/and GP staff subgroup(s): Primary care providers (PCPs) (physician (internal medicine and family practice)) and nurse practitioner Patients with obesity subgroup(s): NA Patients in the study only with obesity or both overweight and obesity included: Only patients with obesity Types of weight‐related issues: Medical co‐morbidities, diabetes, cholesterol, blood pressure	Focus group study	PCPs use a variety of strategies to communicate with their patients about weight loss. A part of PCPs already use patient‐centered approaches to communicate with their patients about weight loss, suggesting that future weight counseling interventions should be tailored to build upon this strength.
Reference number ^41^: Title: “There's always something else”: Patient perspectives on improving the implementation of obesity guidelines in general practice Author(s): D. Mazza, E. McCarthy, N. Singh, M. Carey, L. Turner, M. Harris Date of publication: 19 September 2020 Journal, volume, issue, pages: Obes res clin Pract. Sep–Oct 2020; 14(5):437–442	Describe patient perspectives on the implementation of obesity guidelines in general practice.	Australia	Qualitative	Sample size: 40 Age: Range 46–84 Sex: 22 female and 18 Geographical location: Melbourne GPs or/and GP staff subgroup(s): General practitioners (GPs Patients with obesity subgroup(s): NA Patients in the study only with obesity or both overweight and obesity included: Both patients with overweight and obesity Types of weight‐related issues: Diabetes, cholesterol had gone up, blood pressure was directly related to weight, health	Semi‐structured telephone interviews	It is of great importance to take into account patient perspectives on obesity management in general practice in order to improve health outcomes. This study provides valuable insights into how people living with obesity can be better managed. Interventions should also include strategies to help patients maintain motivation in making lifestyle changes to support healthy weight loss.
Reference number ^42^: Title: Weight management: What patients want from their primary care physicians Author(s): Michael B. Potter, John D. Vu and Mary Croughan‐Minihane Date of publication: Jun 2001 Journal, volume, issue, pages: J Fam Pract 2001 Jun; 50(6), 513–8	To determine the weight management experiences of patients in primary care, and what those patients want from their physicians.	USA	Quantitative	Sample size: 366 Age: BMI <25 mean 38,3, BMI 25–30 mean 43,1 and BMI >30 mean 46,0 Sex: BMI <25 69% women, BMI 25–30 50% women and BMI >30 70% women Geographical location: California, USA GPs or/and GP staff subgroup(s): two primary care practices (9 family Patients with obesity subgroup(s): NA Patients in the study only with obesity or both overweight and obesity included: Both patients with overweight and obesity Types of weight‐related issues: Health risks of obesity, risks of their weight to their health	Survey	Most patients believed they should lose weight. However this is often not discussed during office visits. Many patients want more help with weight management than they are getting from their primary care physicians.
Reference number ^43^: Title: Engagement between patients with obesity and osteoarthritis and primary care physicians: a cross‐sectional survey Author(s): Deborah B. Horn, Christopher Damsgaard, Kathi Earles, Sheba Mathew and Amanda E. Nelson Date of publication: 25 October 2021 Journal, volume, issue, pages: Postgrad Med. 2021 Nov; 133(8):979–987	To map the medical journey of patients with osteoarthritis and obesity by characterizing the roles of health care providers, influential factors, and how treatment decisions are made.	USA	Quantitative	Sample size: 304 patients and 309 healthcare Age: 58,1 mean patients and NA healthcare providers Sex: 64 male and 240 female patients and 226 male, 80 female and 3 other healthcare providers Geographical location: USA GPs or/and GP staff subgroup(s): Primary care physicians (family practice, Internal medicine, general practice) and nurse Practitioner/Physician Assistant Patients with obesity subgroup(s): NA Patients in the study only with obesity or both overweight and obesity included: Only patients with obesity Types of weight‐related issues: Osteoarthritis, effect of health on their overall health	Online survey	As the care coordinator of patients with osteoarthritis and obesity, primary care physicians have a key role in supporting their patients in the treatment journey. Obesity management guidelines can be valuable resources.
Reference number ^44^: Title: Clinical usefulness of brief screening tool for activating weight management discussions in primary cARE (AWARE): A nationwide mixed methods pilot study Author(s): Evan Atlantis, James Rufus John, Paul Patrick Fahey, Samantha Hocking, Kath Peters Date of publication: 28 October 2021 Journal, volume, issue, pages: PLoS One. 2021 Oct 28; 16(10):e0259220	Assess the clinical usefulness of a new screening tool based on the Edmonton obesity staging system (EOSS) for activating weight management discussions in general practice.	Australia	Mixed‐method	Sample size: 30 (5 GPs and 25 patients) Age: <45 (*n*: 6) and >45 (*n*: 19) of patients. GPs NA Sex: 5 male and 20 female of patients. GPs NA Geographical location: Australia GPs or/and GP staff subgroup(s): General practitioners (GPs) Patients with obesity subgroup(s): NA Patients in the study only with obesity or both overweight and obesity included: Both patients with overweight and obesity Types of weight‐related issues: Weight related complications, diabetes, high blood pressure, medical conditions, medical illness, weight‐related health risks, cardiovascular risk factors, stroke, heart attack, swelling of the legs, heart failure, medical complications, medical implications of obesity, high cholesterol	Cross‐sectional and semi‐structured interviews	The EOSS‐2 risk tool is potentially clinically useful for activating weight management discussions in general practice. Further research is required to assess feasibility and applicability.
Reference number ^45^: Title: Patient's experience with comorbidity management in primary care: a qualitative study of comorbid pain and obesity Author(s): E. Amy Janke, Michelle L. Ramirez, Brittany Haltzman, Megan Fritz and Andrea T. Kozak Date of publication: 17 Mar 2015 Journal, volume, issue, pages: Primary Health Care Research & Development 2016; 17: 33–41	Examine perceptions of those with comorbid chronic pain and obesity regarding their experience of comorbidity management in primary care settings.	USA	Qualitative	Sample size: 30 Age: 86%,6% age ≥50 Sex: 80% > male Geographical location: USA GPs or/and GP staff subgroup(s): Primary care providers (physicians, doctor, general medicine) Patients with obesity subgroup(s): Comorbid chronic pain Patients in the study only with obesity or both overweight and obesity included: Both patients with overweight and obesity Types of weight‐related issues: Chronic pain, stress on your heart, all these other good things, blood pressure will go down, comorbid pain or weight symptoms, arthritis, low back pain, osteoarthritis	Semi‐structured interviews	Findings suggest providers should engage in integrated communication regarding weight and pain, targeting this multimorbidity using methods aligned with priorities discussed by patients.
Reference number ^46^: Title: GPs' attitudes, objectives and barriers in counseling for obesity – a qualitative study Author(s): Ulrike Sonntag, Anna Brink, Babette Renneberg, Vittoria Braun & Christoph Heintze Date of publication: 30 Oct 2011 Journal, volume, issue, pages: Eur J Gen Pract. 2012 Mar; 18(1):9–14	Identify GPs' perspectives on counseling patients with overweight and obesity.	Germany	Qualitative	Sample size: 15 Age: 51 average age Sex: 6 male and 9 female Geographical location: Berlin, Germany GPs or/and GP staff subgroup(s): General practitioners (GPs) Patients with obesity subgroup(s): NA Patients in the study only with obesity or both overweight and obesity included: Both patients with overweight and obesity Types of weight‐related issues: High blood pressure, extremely high level of cholesterol, related co morbidities (e.g. knee problems), acute problems (e.g. gastro‐intestinal diseases), musculoskeletal system, risk of diabetes, risk in many organs, cardiovascular risks, obesity‐related diseases	Semi‐structured interviews	Care for obese patients is perceived as ineffective and frustrating. Recommended solutions include further education to improve GPs' communication techniques, for example, to trigger patients’ motivation.
Reference number ^47^: Title: The art and complexity of primary care clinicians' preventive counseling decisions: Obesity as a case study Author(s): Andrew L. Sussman, Robert L. Williams, Robert Leverence, Park W. Gloyd, Benjamin F. Crabtree Date of publication: Jul–Aug, 2006 Journal, volume, issue, pages: Ann Fam Med 2006; 4:327–333	Look for factors that influence clinicians' decisions to include preventive counseling in the brief primary care encounter by using obesity as a case study.	USA	Mixed method	Sample size: 30 in interview/focus group and 146 in survey Age: NA Sex: 13 female interview/focus group and 55% female of survey Geographical location: New Mexico, USA GPs or/and GP staff subgroup(s): Primary care clinicians (family physician, general internists and nurse practitioners and physician assistants) Patients with obesity subgroup(s): NA Patients in the study only with obesity or both overweight and obesity included: Only patients with obesity Types of weight‐related issues: Diabetes, heart disease, arthritis	Survey and in‐depth interviews or analytic focus groups	Clinician decisions to include obesity and other types of preventive counseling in the brief encounter reflect the art and complexity of management Of the encounter. Future efforts to enhance the delivery of preventive counseling Will need to move beyond linear models of behavior change to recognize this complex environment.
Reference number ^48^: Title: A taboo topic? How general practitioners talk about overweight and obesity in New Zealand Author(s): Lesley Gray Maria Stubbe, Lindsay MacDonald, Rachel Tester, Jo Hilder, Anthony C. Dowell Date of publication: 9 May 2018 Journal, volume, issue, pages: J Prim Health Care. 2018 Jun; 10(2):150–158	To identify communication strategies used by GPs to open the topic of weight and weight management in routine consultations.	New Zealand	Qualitative	Sample size: 36 Age: 20–89 Sex: 20 male and 16 female Geographical location: New Zealand GPs or/and GP staff subgroup(s): General practitioners (GPs) Patients with obesity subgroup(s): NA Patients in the study only with obesity or both overweight and obesity included: Both patients with overweight and obesity Types of weight‐related issues: Effect on your blood sugar, blood pressure, bloods have come back totally fine, (medical problem such as cardiovascular health, diabetes or arthritis), health benefits, insulin resistance	Secondary analysis of video‐recorded consultations	The topic of weight was initiated more often by GPs than patients and was raised mostly once or twice in a consultation and occasionally as many as six times. GPs employed opportunistic strategies twice as often as they used structured strategies.
Reference number ^49^: Title: Views of Black women patients with obesity on desired and undesired weight‐focused clinical encounters Author(s): Carolyn M. Tucker, Julia Roncoroni, Kirsten G. Klein, Terry O. Derias. Wafaa Ateyah, Jaime Williams, Nwakaego A Nmezi, Nipa R. Shah, Lori A. Bilello, Stephen Anton Date of publication: 8 June 2021 Journal, volume, issue, pages: Clin Obes. 2021 Oct; 11(5):e12468	To identify the views of black women primary care patients with overweight or obesity regarding Desired and undesired verbal and non‐verbal behaviors by providers In provider‐patient clinical encounters, focused on losing weight, Maintaining weight loss, and/or obesity.	USA	Qualitative	Sample size: 15 Age: 40% age 36% to 64% and 60% 65 or older Sex: Women Geographical location: USA GPs or/and GP staff subgroup(s): Primary care physician Patients with obesity subgroup(s): Black women Patients in the study only with obesity or both overweight and obesity included: Both patients with overweight and obesity Types of weight‐related issues: Health issues, affect heart, heart attack, stroke	Focus group study	Qualitative data yielded five distinct themes: Weight‐focused discussions, desired weight‐focused support, undesired weight‐focused discussions, desired attitudes and behaviors during weight‐focused discussions and building physician‐patient rapport.
Reference number ^50^: Title: Moral discourse in general practitioners' accounts of obesity communication Author(s): Maxine Blackburn, Afroditi Stathi Date of publication: 24 Mar 2019 Journal, volume, issue, pages: Social Science & Medicine (1982), 2019‐06, Vol.230, p. 166–173	Examine the discursive power relations that shape how GPs understand and talk about obesity using a novel methodology To elicit responses from GPs about raising the topic of weight.	United Kingdom	Qualitative	Sample size: 20 Age: 21–60 Sex: 8 male and 12 female Geographical location: South West of England GPs or/and GP staff subgroup(s): General practitioners (GPs) Patients with obesity subgroup(s): NA Patients in the study only with obesity or both overweight and obesity included: Patients with obesity Types of weight‐related issues: Risk factor for a(nother) medical problem, diabetes, bad arthritis in their knees, foot problem, plantar fasciitis, excess weight related to an already established medical problem	Trigger film interviews	The findings suggest that GPs both reproduce and resist moral discourse surrounding body weight. They construct obesity as an individual behavioral problem whilst simultaneously drawing on socio‐cultural discourse which positions Body weight as central to social identity, situating obesity within a context of stigma and positioning patients as powerless to lose weight.
Reference number ^51^: Title: Primary care patient and practitioner views of weight and weight‐related discussion: a mixed‐methods study Author(s): Calum T McHale, Anita H Laidlaw, Joanne E Cecil Date of publication: 9 March 2020 Journal, volume, issue, pages: BMJ Open. 2020 Mar 9; 10(3):e034023	To understand the beliefs that primary care practitioners and patients with overweight and obesity have about obesity and primary care weight management in Scotland	United Kingdom	Mixed method	Sample size: 305 patients and 14 PCPs Age: Patients 18–75+, GPs 18–54, practice nurses 35–54 Sex: Patients 166 female and 139 male, GPs 4 female and 8 male, practice nurses 2 female Geographical location: Scotland, United Kingdom GPs or/and GP staff subgroup(s): 12 general practitioners (GPs) and 2 practice nurses Patients with obesity subgroup(s): NA Patients in the study only with obesity or both overweight and obesity included: Both patients with overweight and obesity Types of weight‐related issues: (Their weight) is an issue where it's clearly impacting on their (health), clinical weight issues, obesity feeds into (their other health issues), patients' excessive weight was impacting directly on their health	Questionnaires and semi‐structured interviews	Incongruent and/or inaccurate beliefs held by primary care practitioners and patient may present barriers to effective weight discussion and management in primary care. There is a need to review, standardize and clarify primary care weight management processes in Scotland Acknowledging a shared responsibility for obesity as a disease may improve outcomes for patients with overweight and obesity

*Note*: Because some of the studies uses the same abbreviations for different concepts, the specific abbreviation used in the different studies will be stated in the table for each study.

Abbreviations: GP/GPs, General practitioner(s); GP staff, General practice staff; NA, Not applicable.

**TABLE 3:** Descriptive numerical analysis and summary of the included studies (modified from “tabular presentation of data for a scoping review,” 11.2.9, JBI Manual for Evidence Synthesis^21^).

Parameter	Results	
Number of types of studies	1. Quantitative studies: 3	
	2. Qualitative studies: 12	
	3. Mixed‐method studies: 5	
Geographical locations represented in the studies and number	USA: 10	
	United Kingdom: 3	
	Canada: 1	
	Norway: 1	
	Germany: 1	
	Australia: 2	
	New Zealand: 1	
	Singapore: 1	
GPs or GP staff subgroups represented and number^a^	GPs: 20	
	GP staff subgroups: 6	
Patient with obesity subgroups and number	Low‐Income African American women who Successfully lost weight: 1	
	Patients with comorbid chronic pain: 1	
	Black women: 1	
Number of studies dealing patients with obesity and/or overweight	Only dealing with patients with overweight: 0	
	Only dealing with patients with obesity: 6	
	Dealing with both patients with overweight and obesity: 14	
Weight‐related issues and number^b^	Impact on general health	18
	Diabetes and prediabetes	15
	Hypertension and impact on blood pressure	14
	Hyperlipidemia and affected blood lipids	8
	Cardiovascular risk and disease	7
	Peripheral edema	2
	Stroke	3
	Urinary problems	1
	Kidney disease	1
	Affected blood tests	2
	Heartburn	1
	Musculoskeletal problems	8
	Obstructive sleep apnea	1
	Depression	1
Number of studies containing information about the subdivisions of communication	When/in which part of the consultation the weight‐related issues are addressed: 1	
	Who initiates the communication about weight‐related issues: 17	
	How the weight‐related issues are addressed: 19	
	How the weight‐related issues are handled: 16	
	Differences in general practitioners' or general practice staff's and patients' views and experiences of communication about weight‐related issues: 1	
	Obstacles or challenges in relation to communication about weight‐related issues: 18	

^a^ The numbers/counts in the table is number of the 20 included studies dealing with GPs and/or GP subgroups.

^b^ The number in the table is number of the 20 included studies dealing with communication about weight‐related issues. The weight related issues in the table are major categories which the authors of this review found most suitable to describe the weight‐related issues mentioned in the included studies.

### 3.2 Quality of the studies

The Quality Assessment Tool for Studies with Diverse Designs (QATSDD)^30^ was used (Table 4). In general, the quality of the included studies, and therefore literature on the topic differed from relatively low quality to relatively high quality. Most of the included studies got about half of the potential quality points. In general, the studies especially got a low score, because of no explicitly stated research question. Also, many of the studies did not mention if the sample size of the study was considered to fit the analytical requirements. Finally, few studies used user involvement in design. On the other hand, the studies got a high score because of statements of an explicit theoretical framework and descriptions of research setting and of procedure for data collection.

**TABLE 4:** Overview of quality assessment of the included studies.

Citation details	Quality rating (score/%)
Malterud and Ulriksen^32^	12/42/28.6%
Lee et al.^33^	28/42/66.6%
Schauer et al.^34^	22/42/52.4%
Aboueid et al.^35^	27/42/66.7%
Scott et al.^36^	15/48/31.3%
Petrin et al.^37^	12/42/28.6%
Leedham‐Green and Wylie^38^	23/42/54.8%
Banerjee et al.^39^	27/48/56.3%
Gudzune et al.^40^	33/42/78.6%
Mazza et al.^41^	21/42/50%
Potter et al.^42^	17/42/40.5%
Horn et al.^43^	16/42/38.1%
Atlantis et al.^44^	30/48/62.5%
Janke et al.^45^	21/42/50%
Sonntag et al.^46^	19/42/45.4%
Sussman et al.^47^	30/48/62.5%
Gray et al.^48^	15/42/35.7%
Tucker et al.^49^	23/42/54.8%
Blackburn and Stathi^50^	29/42/69%
McHale et al.^51^	26/48/54.2%

*Note*: QATSDD uses a 16 item scoring system with a Likert scale (0–3 point) for each item. Some items only apply to quantitative or qualitative studies (highest score possible for qualitative and quantitative 42 points). Mixed method studies qualifies for both quantitative and qualitative quality assessment and therefore has a total possible score of 48. The score of each study out of total possible and also in percentage of total possible can be viewed in the table.

### 3.3 Communication about weight‐related issues

This section of the results will present the different parts of the communication about weight‐related issues. An overview of the included articles with information about content of examples of the different subcategories of communication is presented in Table 5. In the following headings the most frequent themes of the extracted examples from the studies will be presented and gathered with quotations to the different included studies, giving an insight of the diversity of the difficult and complex field of communication about weight‐related issues in general practice.

**TABLE 5:** Overview of examples of the different subcategories of communication in the included studies.

Citation details	When/in which part of the consultation the weight‐related issues are addressed^a^	Who initiates the communication about weight‐related issues	How the weight‐related issues are addressed	How the weight‐related issues are handled	Differences in general practitioners' or general practice staff's and patients' views and experiences of communication about weight‐related issues	Obstacles or challenges in relation to communication about weight‐related issues
Malterud and Ulriksen^32^	During consultations/not specified^b^	Few examples^c^	Few examples	Few examples	Only the patients' perspectives/not applicable	Many examples^d^
Lee et al.^33^	During consultations/not specified	Few examples	Many examples	Many examples	Only the primary care physicians' perspectives/not applicable	Few examples
Schauer et al.^34^	During consultations/not specified	Many examples	Many examples	Many examples	Only the clinicians' perspectives/not applicable	Many examples
Aboueid et al.^35^	During consultations/not specified	Few examples	Many examples	Few examples	Only the health professionals' perspectives/not applicable	Few examples
Scott et al.^36^	During consultations/not specified	Many examples	Many examples	Few examples	Not mentioned	Not mentioned
Petrin et al.^37^	During consultations/not specified	Many examples	Many examples	Not mentioned	Only the health care professionals'' perspectives/not applicable	Few examples
Leedham‐Green and Wylie^38^	During consultations/not specified	Few examples	Not mentioned	Few examples	Not mentioned	Many examples
Banerjee et al.^39^	During consultations/not specified	Few examples	Few examples	Few examples	Only the patients' perspectives/not applicable	Few examples
Gudzune et al.^40^	During consultations/not specified	Few examples	Few examples	Few examples	Only the primary care providers' perspectives/not applicable	Many examples
Mazza et al.^41^	Few examples	Few examples	Many examples	Few examples	Only the patients' perspectives/not applicable	Few examples
Potter et al.^42^	During consultations/not specified	Not mentioned	Few examples	Few examples	Only the patients' perspectives/not applicable	Few examples
Horn et al.^43^	During consultations/not specified	Not mentioned	Many examples	Many examples	Many examples	Many examples
Atlantis et al.^44^	During consultations/not specified	Many examples	Many examples	Many examples	Not mentioned	Few examples
Janke et al.^45^	During consultations/not specified	Many examples	Many examples	Many examples	Only the patients' perspectives/not applicable	Many examples
Sonntag et al.^46^	During consultations/not specified	Many examples	Many examples	Few examples	Only the general practitioner's perspectives/not applicable	Many examples
Sussman et al.^47^	During consultations/not specified	Few examples	Few examples	Not mentioned	Only the clinicians' perspectives/not applicable	Many examples
Gray et al.^48^	During consultations/not specified	Many examples	Many examples	Many examples	Only the general practitioner's perspectives/not applicable	Not mentioned
Tucker et al.^49^	During consultations/not specified	Not mentioned	Few examples	Few examples	Only the patients' perspectives/not applicable	Many examples
Blackburne and Stathi^50^	During consultations/not specified	Few examples	Many examples	Not mentioned	Only the general practitioner's perspectives/not applicable	Many examples
McHale et al.^51^	During consultations/not specified	Few examples	Many examples	Not mentioned	Not mentioned	Many examples

^a^ The headings of columns of this table is the original division of subcategories when communicating obesity related to the review question—also mentioned in the online available review protocol.

^b^ During consultations/not specified: in all the included studies communication about weight‐related issues occurred, but when is often not specified—for example, when taking anamnesis, making the clinical examination or when talking about the treatment of the patient's symptoms.

^c^ Few examples: one to two examples of the communication subcategory in the included study. Marked with light gray.

^d^ Many examples: >2 examples of the communication subcategory in the included study. Marked with darker gray.

### 3.4 Timing for addressing weight‐related issues

Only one study held information regarding when the weight‐related issues are addressed in general practice^41^ (Table 5). This example was referring to the topic addressed after a blood test, where a patient with obesity said: “I did a blood test and found out that my cholesterol had gone up… That is when we spoke about the weight issues.”^41^

### 3.5 Initiating communication about weight‐related issues

Most of the studies held few or many examples of who initiates the communication of the weight‐related issues in general practice. This included both examples of the general practitioners and general practice staff subgroup and the patients initiating communication of the topic^32^, ^33^, ^34^, ^35^, ^36^, ^37^, ^38^, ^39^, ^40^, ^41^, ^44^, ^45^, ^46^, ^47^, ^48^, ^50^, ^51^ (Table 5). All of these studies held information of the general practitioner or general practice staff subgroup initiating the discussions^32^, ^33^, ^34^, ^35^, ^36^, ^37^, ^38^, ^39^, ^40^, ^41^, ^44^, ^45^, ^46^, ^47^, ^48^, ^50^, ^51^ but also some studies held information of the patient initiating the discussions.^36^, ^37^, ^38^, ^41^, ^45^, ^48^

### 3.6 Addressing weight‐related issues

All studies but one held information on how the weight‐related issues are addressed^32^, ^33^, ^34^, ^35^, ^36^, ^37^, ^39^, ^40^, ^41^, ^42^, ^43^, ^44^, ^45^, ^46^, ^47^, ^48^, ^49^, ^50^, ^51^ (Table 5). Some studies held general examples of positive ways to address the topic. As an example the topic could be addressed by discussing the risks of developing or having weight‐related issues as a motivating factor for change and improvement of risk factors for the patients' health.^34^, ^35^, ^36^, ^39^, ^40^, ^41^, ^45^, ^47^, ^48^, ^49^, ^50^ In addition, some studies mentioned the communication of weight‐related issues in general practice as having focus on general health and wellness rather than weight loss.^40^, ^46^, ^47^, ^51^ On the other hand, many studies also documented the negative experience a discussion of weight‐related issues in general practice can be. This could for example, be that the issue of the patients' complaints was generally attributed to weight by the primary care provider, irrespective of the specific cause.^32^, ^50^ Likewise, some studies mentioned the central topic of stigma in relation to the weight‐related conversations.^44^, ^50^ Also, some studies highlighted the view of raising the issue as an general practitioner obligation.^50^, ^51^ In addition to the above mentioned themes, primary care providers used direct language in different manners in the included studies,^39^, ^45^, ^46^, ^49^ for example, in a paternalistic way of addressing the issue to the patient: “You got an atomic bomb here. Now you go figure it out.”^39^ and “… then I would tell him: ‘It won't work like this! Something has to change!’ (GP14).”^46^ Some studies also highlighted the reference to possible family history of chronic conditions/weight‐related issues as a way of addressing the topic.^36^, ^44^, ^48^

Finally, one study used a screening tool for estimating patient risk of having weight‐related health problems.^44^

### 3.7 Handling weight‐related issues

Many studies held information of how the weight‐related issues are handled^32^, ^33^, ^34^, ^35^, ^36^, ^38^, ^39^, ^40^, ^41^, ^42^, ^43^, ^44^, ^45^, ^46^, ^48^, ^49^ (Table 5).

Firstly, many of the included studies held information of how weight‐related issues were handled in general practice, for example, the primary care provider giving general recommendations regarding weight loss,^32^, ^33^, ^34^, ^36^, ^40^, ^42^, ^43^, ^44^, ^45^, ^46^ offering additional weight counseling^36^, ^46^ or giving informational handouts or using standardized messages or material.^39^, ^40^, ^41^, ^43^, ^45^ In addition, in some studies the providers gave specific advice on behavior changes or lifestyle changes,^39^, ^43^, ^49^ exercise^33^, ^42^, ^43^, ^48^, ^49^ and diet modification.^33^, ^34^, ^35^, ^41^, ^43^, ^45^, ^48^, ^49^ Providers also used changes of or addition of medications.^36^, ^40^, ^43^ Lastly, also the use of referral to specialists was used as tools to handle weight‐related issues in general practice, for example, referral for diabetes group therapy or an educational program,^32^, ^34^ external weight management programs or local resources,^33^, ^34^, ^42^, ^44^ dieticians or nutritionists,^33^, ^38^, ^39^, ^43^, ^45^ chronic disease nurse counselors or practice nurses,^33^, ^38^ obesity medicine specialist or referral or encourage to bariatric surgery.^33^, ^43^

Differences in general practitioners' or general practice staffs' and patients' views and experiences of communication about weight‐related issues.

Only one study held information on this topic^43^ (Table 5): “When talking about weight or weight management with their patients who have osteoarthritis and obesity, primary care physicians were much more likely than patients to report discussing the effect patients’ weight has on their osteoarthritis and overall health, helping patients set goals to improve their weight and understand why they have excess weight, and making patients aware of medications that will help them lose weight….”^43^

### 3.8 Obstacles and challenges in relation to communication about weight‐related issues

Most studies held information on obstacles or challenges in relation to communication on this topic^32^, ^33^, ^34^, ^35^, ^37^, ^38^, ^39^, ^40^, ^41^, ^42^, ^43^, ^44^, ^45^, ^46^, ^47^, ^49^, ^50^, ^51^ (Table 5). Overwhelmingly, it was the primary care provider who was seen as an obstacle for communication on this topic. One major obstacle mentioned by the studies was lack of time.^37^, ^38^, ^40^, ^41^, ^43^, ^44^, ^46^, ^47^, ^51^ Also, one consistent topic was primary care providers looking down on patients and thinking they were not willing to improve or that they lacked motivation.^32^, ^38^, ^43^, ^46^, ^47^, ^49^, ^51^ In relation to this, the primary care providers often informed the patients of their weight status but did not give additional information.^39^, ^45^, ^50^ In addition, primary care providers could show signs of disapproval.^32^, ^45^, ^49^ The providers generally found it difficult to shift the attention to the weight or raise the issue in general,^33^, ^38^, ^44^, ^50^ for example, in acute situations.^34^ Sometimes the providers argued that patients already were aware that they had obesity but were not interested in starting a conversation about it.^44^, ^51^ Also, the primary care provider felt the patients did not present obesity as the main cause of the medical condition.^44^, ^50^, ^51^ Primary care providers could perceive communication on this topic as a risk of offending new patients in the clinic^34^, ^35^, ^46^ or offending the patients in the clinic in general.^38^, ^51^ Sometimes the primary care provider's own weight was perceived as an obstacle or having an impact on the conversation.^35^, ^50^ Finally, the providers found that they lacked training in obesity management,^37^, ^51^ had a general lack of knowledge and skills regarding how to address obesity,^40^, ^51^ for example, before morbidity sets in.^38^ Finally, primary care providers felt a lack of treatment possibilities, and missing material on the topic^46^, ^50^, ^51^

## 4 DISCUSSION

The objective of this review was to identify what is known about communication about weight‐related issues in general practice with adult patients with obesity. This led to inclusion of 20 relevant studies and identification of the key concepts of the available peer‐reviewed studies and knowledge gaps on this topic. Overall, this review reveals increasing research on the topic during the recent 8 years. The studies primarily had a qualitative study design and were conducted in USA. All of the included studies dealt with general practitioners and very few studies focused on a specific patient subgroup with obesity. The most frequent major categories of weight‐related issues were “impact on general health,” “diabetes and prediabetes,” “hypertension and impact on blood pressure,” “hyperlipidemia and affected blood lipids” and musculoskeletal problems. Only one study mentioned psychosocial weight‐related issues and none mentioned cancer as topics of discussion. The studies were in general of medium quality when doing quality assessment. As regard the topic of communication, this scoping review found multiple examples regarding the “who initiates”‐, “how they are addressed”‐, “how are they handled”‐ and “obstacles/challenges”‐sub‐questions. Few studies held information regarding “when they are addressed” and “differences in views.” As mentioned above, this review gathered plenty of information regarding who initiates communication about weight‐related issues in general practice. All of the included studies mentioned the general practitioner or general practice staff initiating the discussions. Only six of the studies showed the patient initiating the discussions. One of the included studies had initiation of weight discussions as main focus stating general practitioners more often than patients initiates these discussions and clinical relevance as the most notable way of achieving a constructive dialog which fits the findings of this review.^48^ This review included many studies demonstrating how the weight‐related issues are addressed in general practice with great diversity. Firstly, many of the included studies held information of using weight‐related issues as a motivating factor for patient change and reduction of risk on one's health. One newly published study using the ACTION‐IO data for analysis identified the significantly associated variable with motivated people with obesity as “important goal as part of weight management: To reduce the risks associated with excess weight/prevent a health condition.”^52^ This emphasized the use and importance of the use of weight‐related issues as a motivator when discussing this topic in general practice. Secondly, some studies in this scoping review held information on the primary care provider using direct language, including use of a paternalistic way of addressing the weight‐related issues. In the studies the patients also argued discomfort with direct paternalistic language. Other literature also highlighted the importance of patient centered care using for example, shared decision‐making instead of paternalism.^53^ Lastly, the authors of this article found one new pilot study explaining the use of a screening tool for patients having weight‐related issues.^44^ Also, this review included many studies with information on how weight‐related issues are handled. In general, this review highlighted the already known use of general recommendations of weight loss or more specific advice about exercise or diet modifications. This kind of intervention seems to induce clinically meaningful weight loss in the literature.^54^ This scoping review also showed a great use of referral to specialists, for example, weight management programs which seems to be an effective way depending on the duration.^55^ Few of the studies included referral to bariatric surgery. However, it is important to underline the effectiveness of this way of severe obesity management.^56^ The last subdivision of communication was obstacles and challenges in relation to communication about weight‐related issues with patients with obesity in general practice. One great obstacle reported was lack of time, which is also mentioned as an important barrier for effective obesity care in other literature.^57^ This scoping review also found a great diversity of ways in which the primary care providers have biased a general negative view of and impression of the patients. The before mentioned supports the great amount of literature about stigmatization of patients with overweight and obesity in health care,^11^ which also tends to have a negative effect on health behavior.^58^ Finally, the providers missed training and knowledge on obesity communication. This is also known in the literature where the use of motivational interviewing or the 5 As framework is known but still poorly understood as to the effect on patient outcomes.^59^ One major knowledge gap identified when conducting this scoping review was what despite the fact that identifying 20 studies containing information on communication about weight‐related issues with adult patients with obesity in general practice, not one of the included studies had this topic as its main focus. This also shows in the titles and aims of the included studies in Table 2 and also the fact that the concept of “many examples” in Table 5 of this scoping review only needed to be minimum three examples in the included studies. To ensure the validity on the research in this area, future studies need to focus more exclusively on this topic. A surprising and major finding of this study, was that only one study held information on discussion of psychosocial issues—more precisely depression. Furthermore, none of the studies mentioned cancer in the weight‐related discussions even though it has been known for many years as a co‐morbidity related to overweight and obesity.^4^ The main focus of the included studies was clearly the physical weight‐related issues of patient with obesity. This calls for future research to also highlight the topic of psychosocial issues of patients with obesity, for example, depression, anxiety, eating disorders, quality of live etc. Also, future research need to focus on the relation between cancer and overweight and obesity. As mentioned earlier in the discussion, primary care providers mentioned in multiple of the included studies lack of time as a major barrier for initiating and shifting the attention to communication with patients on this topic. One study interestingly did a pilot study of a risk tool of patients having weight‐related issues and mentioned the effectiveness of using such a tool.^44^ More research on this topic is needed to explore ways to decrease stigma in patient‐provider communication in general practice where lack of time is a central theme. Finally, insights into both the patients' and primary care providers' views of this specific encounter in a reasonable time after the encounter is needed, which could be studied through direct observation of the clinical encounter. This would lead to a more nuanced insight into the clinical encounter with minimal risk of recall bias and also make way for a more precise understanding of differences in the patients' and primary care providers' views on this topic. This scoping review is presumably the first study making a clarification of what is known about communication about weight‐related issues in general practice with adult patients with obesity, gathering key concepts and knowledge gaps for researches of interest and future research. The methodology of this scoping review took the description of conduction of scoping reviews by Joanna Briggs Institute^21^ as starting point and followed the five stages of conduction^22^, ^23^ which was performed systematically and stringently as described in the protocol developed a priori. A major search string was developed in corporation with a health science librarian which led to the narrowing from 4862 studies to 20 included studies after full screening done independently by two of the reviewers leading to agreement. Also this review examined a great diversity of data extracted (Table 3), providing a considerable overview of the existing literature on the topic. On the other hand, this scoping review did not include gray literature as scoping reviews often but not always include. However, the aim of this scoping review was to explore the peer‐reviewed published literature to explore the higher quality scientific articles on this topic. This scoping review identified twenty peer‐reviewed qualitative, quantitative, and mixed‐method studies concerning communication about weight‐related issues with adult patients with obesity in general practice. The studies mostly had an acceptable quality. The studies held almost no information on when the weight‐related issues are addressed, and if there were differences general practitioners' or general practice staff's and patients' views and experiences when discussing the topic. Many of the studies held information about who initiates the discussion, how the weight‐relate d issues are addressed and handled and also obstacles and challenges when discussing the topic. Even though 20 studies on the topic were identified, none of the studies had the topic of “weight‐related issues” as a central theme, and therefore future research needs to make this a main focus to one or multiple of the sub‐questions regarding communication of this scoping review. The included studies in general focused on physical weight‐related issues and neglected the psychosocial weight‐related issues—this also need to be implemented in future research. Finally, future research on this topic need to focus on the differences in primary care provider and patient view of discussing the topic for example, making direct observations immediately followed by interviews of both primary care provider and patient.

## CONFLICT OF INTEREST STATEMENT

The authors declare no conflict of interest.
